# *Clavibacter nebraskensis* causing Goss's wilt of maize: Five decades of detaining the enemy in the New World

**DOI:** 10.1111/mpp.13268

**Published:** 2022-09-18

**Authors:** Ebrahim Osdaghi, Alison E. Robertson, Tamra A. Jackson‐Ziems, Hamid Abachi, Xiang Li, Robert M. Harveson

**Affiliations:** ^1^ Department of Plant Protection, College of Agriculture University of Tehran Karaj Iran; ^2^ Department of Plant Pathology, Entomology and Microbiology Iowa State University Ames Iowa USA; ^3^ Department of Plant Pathology University of Nebraska–Lincoln Lincoln Nebraska USA; ^4^ Canadian Food Inspection Agency Charlottetown Laboratory Charlottetown Prince Edward Island Canada; ^5^ Panhandle Research & Extension Center University of Nebraska Scottsbluff Nebraska USA

**Keywords:** actinobacteria, corn, coryneform bacteria, *Microbacteriaceae*, Poaceae, quarantine pathogen, *Zea mays*

## Abstract

Goss's bacterial wilt and leaf blight of maize (*Zea mays*) caused by the gram‐positive coryneform bacterium *Clavibacter nebraskensis* is an economically important disease in North America. *C. nebraskensis* is included within the high‐risk list of quarantine pathogens by several plant protection organizations (EPPO code: CORBMI), hence it is under strict quarantine control around the world. The causal agent was reported for the first time on maize in Nebraska (USA) in 1969. After an outbreak during the 1970s, prevalence of the disease decreased in the 1980s to the early 2000s, before the disease resurged causing a serious threat to maize production in North America. The re‐emergence of Goss's wilt in the corn belt of the United States led to several novel achievements in understanding the pathogen biology and disease control. In this review, we provide an updated overview of the pathogen taxonomy, biology, and epidemiology as well as management strategies of Goss's wilt disease. First, a taxonomic history of the pathogen is provided followed by symptomology and host range, genetic diversity, and pathogenicity mechanisms of the bacterium. Then, utility of high‐throughput molecular approaches in the precise detection and identification of the pathogen and the management strategies of the disease are explained. Finally, we highlight the role of integrated pest management strategies to combat the risk of Goss's wilt in the 21st century maize industry.

**Disease symptoms:**

Large (2–15 cm) tan to grey elongated oval lesions with wavy, irregular water‐soaked margins on the leaves. The lesions often start at the leaf tip or are associated with wounding caused by hail or wind damage. Small (1 mm in diameter), dark, discontinuous water‐soaked spots, known as “freckles”, can be observed in the periphery of lesions. When backlit, the freckles appear translucent. Early infection (prior to growth stage V6) may become systemic and cause seedlings to wilt, wither, and die. Coalescence of lesions results in leaf blighting.

**Host range:**

Maize (*Zea mays*) is the only economic host of the pathogen. A number of Poaceae species are reported to act as secondary hosts for *C. nebraskensis*.

**Taxonomic status of the pathogen:**

Class: *Actinobacteria*; Order: *Micrococcales*; Family: *Microbacteriaceae*; Genus: *Clavibacter;* Species: *Clavibacter nebraskensis.*

**Synonyms:**

*Corynebacterium nebraskense* (Schuster, 1970) Vidaver & Mandel 1974; *Corynebacterium michiganense* pv. *nebraskense* (Vidaver & Mandel 1974) Dye & Kemp 1977; *Corynebacterium michiganense* subsp. *nebraskense* (Vidaver & Mandel 1974) Carlson & Vidaver 1982; *Clavibacter michiganense* subsp. *nebraskense* (Vidaver & Mandel 1974) Davis et al. 1984; *Clavibacter michiganensis* subsp*. nebraskensis* (Vidaver & Mandel 1974) Davis et al. 1984.

**Type materials:**

ATCC 27794^T^; CFBP 2405^T^; ICMP 3298^T^; LMG 3700^T^; NCPPB 2581^T^.

**Microbiological properties:**

Cells are gram‐positive, orange‐pigmented, pleomorphic club‐ or rod‐shaped, nonspore‐forming, nonmotile, and without flagella, approximately 0.5 × 1–2.0 μm.

**Distribution:**

The pathogen is restricted to Canada and the United States.

**Phytosanitary categorization:**

EPPO code CORBNE.

## Graphical abstract

In this review, we provide an updated overview of the pathogen taxonomy, biology, and epidemiology as well as management strategies of the Goss's wilt disease.

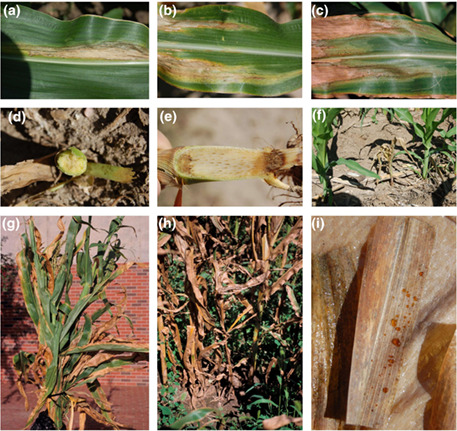

## 1 TAXONOMIC HISTORY OF THE PATHOGEN

A previously unreported bacterial disease was observed on maize (*Zea mays*) in Dawson County in south‐central Nebraska in late August 1969 (Schuster, 1970). The disease was named “leaf freckles and wilt” based on characteristics of the observed symptoms. Preliminary investigations showed a coryneform bacterium was associated with the disease. The causal agent was named *Corynebacterium nebraskense* (Vidaver & Mandel, 1974; Wysong et al., 1973). In the early 1970s, the disease was called Nebraska leaf freckles and wilt, bacterial leaf blight and wilt, bacterial leaf freckles and wilt until Goss's bacterial wilt and blight was selected as the preferred common name (Schuster, 1975).

Until the 1970s, coryneform plant‐pathogenic bacteria infecting different plants were classified in separate species. A taxonomic study on plant‐pathogenic *Corynebacterium* species showed the coryneform species were a relatively homogeneous group of organisms and should not be divided into 13 species (Dye & Kemp, 1977). Consequently, it was suggested all species should form a single species, *Corynebacterium michiganense*. However, plant‐pathogenic coryneform species could be divided into several groups when differences are detectable by cultural, biochemical, or serological methods. Thus, the maize pathogen was classified as *Corynebacterium michiganense* pv. *nebraskense*. Still others proposed to exclude plant‐pathogenic coryneform species from *Corynebacterium* (Lazar, 1968; Lelliott, 1974). Several years later, the genus *Clavibacter* was described (Davis et al., 1984), which contained almost all phytopathogenic coryneform bacteria, and the Goss's wilt pathogen was reclassified as *Clavibacter michiganense* subsp. *nebraskense* (Davis et al., 1984). In the subsequent years, according to the nomenclature rules of bacterial taxonomy, the name was revised to *Clavibacter michiganensis* subsp. *nebraskensis*, and four other plant‐pathogenic subspecies were described within the species. Single locus‐based phylogenetic analyses confirmed the close taxonomic relationship of the Goss's wilt pathogen with the four other subspecies (Lee et al., 1997).

**FIGURE 1:**
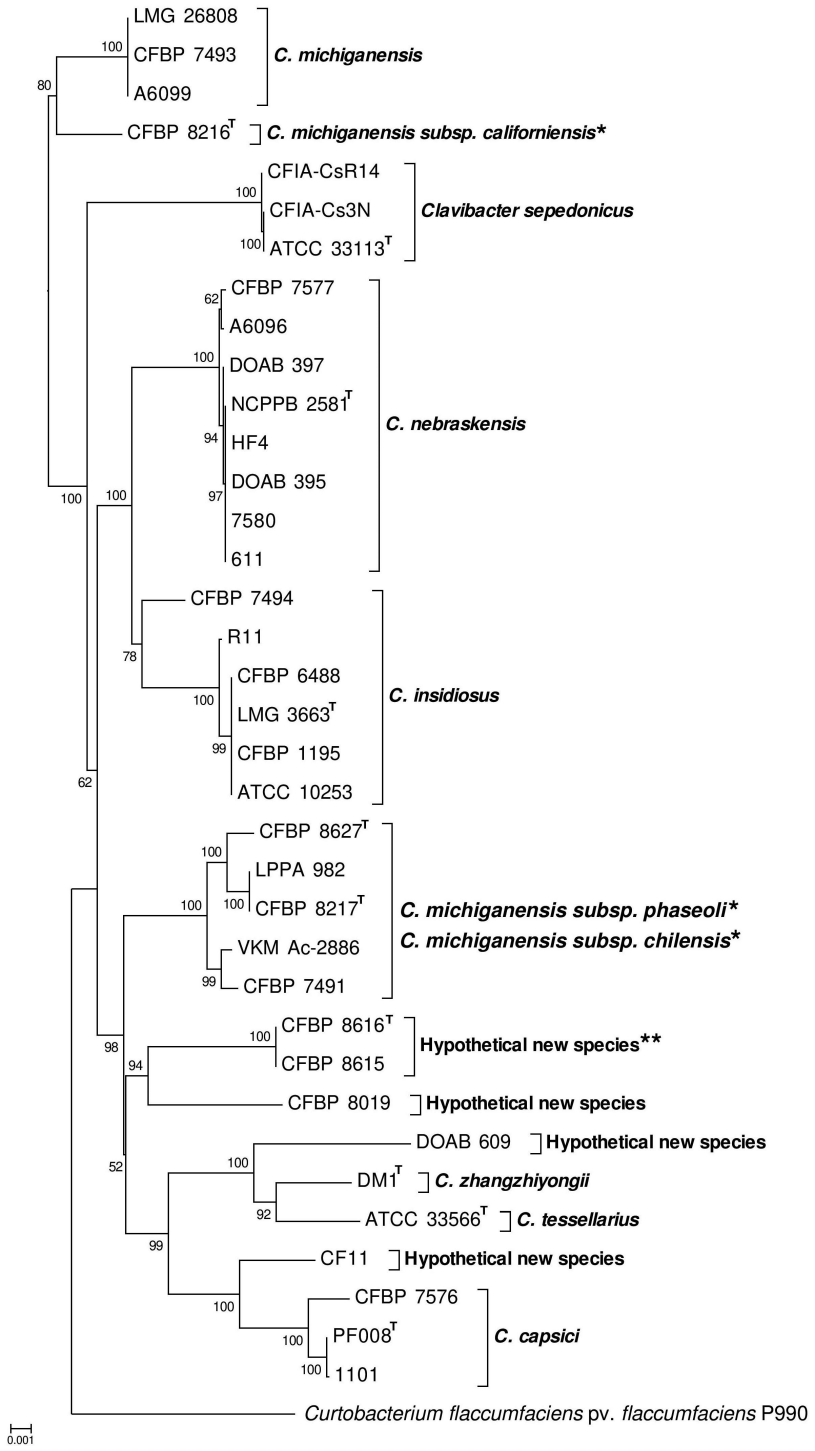
Whole‐genome sequence‐based phylogenetic analysis of plant‐pathogenic members of *Clavibacter*. The neighbour‐joining tree was generated based on the whole‐genome sequences of 49 *Clavibacter* strains using the galaxy Europe online service (https://galaxyproject.eu/) and 1000 bootstrap replications. *Clavibacter nebraskensis* strains were clustered in a monophyletic clade phylogenetically related to the alfalfa pathogen *C. insidiosus*. *Needs taxonomic re‐evaluation. **Taxonomic description is in preparation

Most recently a reclassification of *Clavibacter* spp. into six new species was proposed based on genomic information such as average nucleotide identity (ANI) and digital DNA–DNA hybridization (dDDH) indices (Li et al., 2018). The original subspecies of *C. michiganensis* sensu lato were elevated to the species level, thus the Goss's wilt pathogen was designated as *Clavibacter nebraskensis*. Further complete genome sequence‐based investigations, that is, comparative genomics and phylogenetic analyses using all the publicly available genome sequences of the genus, have confirmed the new taxonomic changes and show *C. nebraskensis* is a monophyletic taxon encompassing only the maize pathogenic strains of the genus (Osdaghi et al., 2018a, 2020a). *C. nebraskensis* is phylogenetically closely related to the alfalfa wilt pathogen *C. insidiosus* (Figure 1). The Goss's wilt pathogen along with the alfalfa wilt pathogen and the wheat bacterial mosaic pathogen *C. tessellarius* are considered Nebraska natives (Harveson, 2015).

## 2 GEOGRAPHIC DISTRIBUTION AND MID‐2000s RESURGENCE OF THE DISEASE

Goss's wilt and leaf blight became a serious and destructive bacterial disease throughout the 1970s after its first report in 1969 from south‐central Nebraska. Over the next decade, it was identified on samples from at least 53 other counties within Nebraska and subsequently confirmed in the surrounding states of Iowa, South Dakota, Kansas, and Colorado (Schuster et al., 1972; Wysong et al., 1973). The pathogen eventually spread to most of the US corn‐growing states before nearly disappearing in the mid‐1980s just as suddenly as it first appeared (Harveson, 2015; Jackson et al., 2007; Mbofung et al., 2016; Robertson & Jesse, 2008). In 2004, the disease suddenly and unexpectedly re‐emerged in approximately one dozen fields in north‐east Colorado and western Nebraska before becoming particularly severe in 2005 and 2006. In 2006 alone, the pathogen was confirmed on samples from more than 50 fields with many more suspected, but unsubstantiated, reports being made (Jackson et al., 2007). The majority of these outbreaks in the mid‐2000s occurred in western Nebraska, north‐east Colorado, and south‐east Wyoming, where it continued as the most serious and predominant disease of maize in the central High Plains (Harveson, 2020). During the 2007–2008 seasons, the disease presence in western Nebraska decreased. At the same time, an increase in incidence and severity was detected in eastern and central Nebraska, where the disease had not been reported since the late 1970s. Goss's wilt and leaf blight was confirmed in maize samples from 15 and 24 counties in 2008 and 2009, respectively. During 2009, a sharp increase in the number of wilt‐infested fields was observed in many additional counties of eastern Nebraska, representing the first documentation of the disease being found statewide in more than two decades (Jackson et al., 2007). In addition, the disease spread to and developed in other Midwest states, including for the first time Indiana (Ruhl et al., 2009).

During 2010 and 2011, the disease was particularly destructive across the midwestern corn belt states with new reports emerging from the widely separated states of Louisiana (Singh et al., 2015), Minnesota (Malvick et al., 2010), Missouri (Hosack et al., 2016; Sweets & Hosack, 2014), North Dakota (Friskop et al., 2014), Texas (Korus et al., 2011), and Alberta, Canada (Howard et al., 2015). Although the disease was observed in Ontario, Canada, during the 1999 growing season, subsequent annual surveys in Ontario and Quebec did not detect the pathogen (Zhu et al., 2005). The limited distribution of the pathogen in North America since it was first reported in Nebraska in 1969 was suggested to be due to inefficient seed transmission (Biddle et al., 1990; Block et al., 2019). The re‐emergence of the Goss's wilt pathogen is thought to be favoured by a number of factors, including new cultural practices over the last 30 years, such as continuous cropping, reduced tillage, and dramatic acreage increases with overhead sprinkler irrigation (Harveson, 2015). These cultural practices were widely adopted in central and western Nebraska roughly a decade before the sudden and widespread re‐occurrence of the maize pathogen (Harveson, 2015; Jackson et al., 2007). The return of Goss's wilt may also be explained by the discontinuance of growing disease‐resistant cultivars. After the disease seemingly disappeared 30 years ago, producer demand for Goss's wilt‐resistant hybrids waned and many seed companies reduced screening efforts for resistance. In 2005, coinciding with the new outbreaks, less than 25% of seed companies in Nebraska provided disease ratings of their hybrids. Growers impacted by the disease after its re‐emergence sought maize with seed that possessed high levels of disease resistance to Goss's wilt, thus seed companies returned to routine testing and breeding of new resistant hybrids (Jackson et al., 2007; Langemeier et al., 2017). Figure S1 illustrates the distribution of the pathogen in North America as inferred from the EPPO Global Database (https://gd.eppo.int/taxon/CORBNE).

Lastly, the climatic patterns observed throughout the region over this same period could also conjecturally contribute to the re‐emergence. The mid‐2000s were characterized by warmer winters and an extended drought with increasingly higher summer temperatures during the growing season. These factors could have easily improved conditions for pathogen overwintering survival and increased plant stresses (see below: Pathogen Biology; Harveson, 2020; Jackson et al., 2007). Both are well recognized to favour disease development and increased severity for the disease. These observations have also been used to explain the almost simultaneous re‐emergence of a similar, distantly related, gram‐positive bacterial pathogen *Curtobacterim flaccumfaciens* pv. *flaccumfaciens*, which causes wilt disease of dry beans (Harveson, 2020; Harveson et al., 2015; Osdaghi et al., 2020b). Furthermore, the prevalence of bacterial diseases in the region is noteworthy. Bacterial leaf streak (*Xanthomonas vasicola* pv. *vasculorum*) of maize was reported in Nebraska a few years later for the first time in the United States (Hartman et al., 2020; Korus et al., 2017; Ortiz‐Castro et al., 2020). In addition, other bacterial diseases such as bacterial stalk rot (*Pectobacterium carotovorum*) of maize and bacterial leaf streak of wheat (*Xanthomonas translucens* pv. *undulosa*) and others continue to plague the region (Khojasteh et al., 2019; Sapkota et al., 2020).

## 3 SYMPTOMS AND SIGNS OF GOSS'S WILT

**FIGURE 2:**
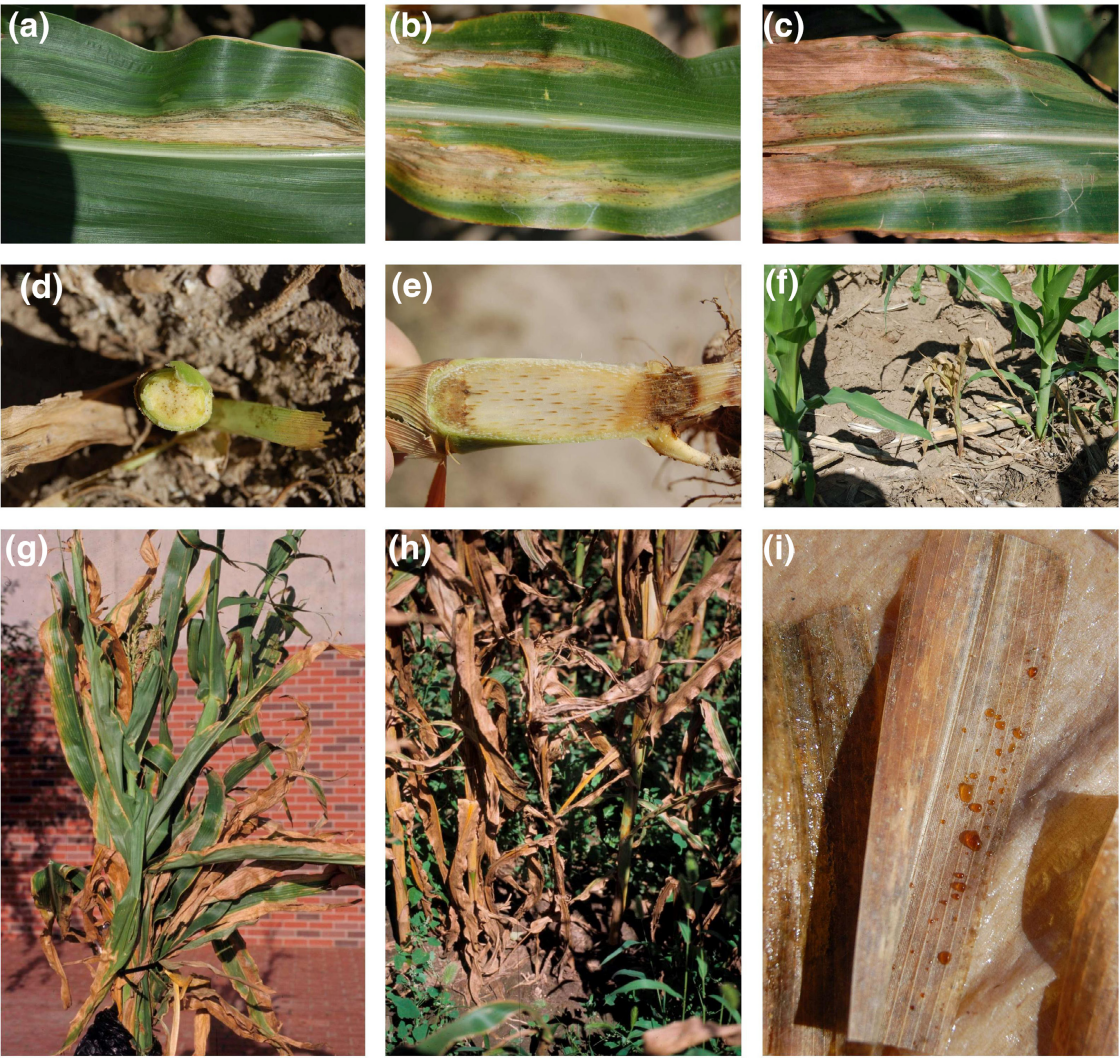
Field symptoms of Goss's wilt caused by *Clavibacter nebraskensis* on aerial parts of maize plants. Aerial symptoms include large, tan‐to‐grey elongated oval leaf lesions (a) that run parallel to the leaf veins (b), which could result in severe blighting or plant death (c). Bacterial colonization of stems during systemic infection can be identified as orange discolouration that may darken to brown or black (d) and be slimy as the infection progresses (e). Early infection of seedlings may result in wilt and plant death (f). While the leaf blight phase may occur at any stage of growth (g), the wilt phase of the disease is less common and usually occurs on severely blighted plants (h). The pathogen produces an extracellular polysaccharide exudate that can ooze out of infected leaf tissue and frequently is found on the surface of infected leaves (i)

Two disease phases are associated with *C. nebraskensis* infection: a foliar blight phase and a systemic wilt phase (Figure 2). Necrotic lesions of the leaf blight phase kill the leaf tissue, reducing photosynthetic areas (Figure 2a–c), while the wilt phase results in systemic movement of the pathogen and often mortality of plants (Figure 2d–i; Wise et al., 2010). Maize plants may be infected by *C. nebraskensis* at any growth stage. Seedlings are more susceptible to infection than older plants. Early (prior to growth stage V6) infection of susceptible genotypes may result in wilt and plant death (Figure 2f; Calub et al., 1974), while also serving as a point source for additional infections. Infection of older plants usually results in leaf blight, the symptoms of which are large tan‐to‐grey elongated oval leaf lesions that run parallel to the leaf veins. Leaf blight lesions on maize leaves of susceptible hybrids are large (6 to >15 cm in length), initially light green to yellow, and quickly become necrotic (Jackson et al., 2007). On resistant hybrids, lesions are smaller (2–6 cm) and reddish. The margins of the lesions are wavy or irregular, water‐soaked and can cover up to 50% of the leaf surface. Eventually, the coalescence of stripes results in a leaf scorch, reminiscent of the effects of drought (Figure 2g,h; Schuster, 1975). Dark‐green‐to‐black water‐soaked spots, known as “freckles”, are visible within the periphery of the lesions (Howard et al., 2015; Schuster, 1975). Bacterial streaming from the cut edge of lesions is visible with light microscopy (Malvick et al., 2010). Symptoms of Goss's wilt after artificial inoculation may vary depending on plant age and inoculum concentration (Calub et al., 1974). *C. nebraskensis* produces an extracellular polysaccharide exudate that can ooze out of infected leaf tissue and is frequently found on the surface of infected leaves (Bauske & Friskop, 2021). The exudate is usually colourless and can be recognized as a shiny or glossy sheen on the leaf surface (Figure 2i). The leaves of infected seedlings may stick together due to exuding bacteria while rapid collapse of those plants with wilt is prevalent. In older plants, this may prevent emergence of tassels and cause the stalk to bend in the form of a loop or form a “buggy whip”. Bacterial colonization of stems during systemic infection plugs the xylem tissue and can be identified as orange discolouration that may darken in colour to brown or black and be slimy as the infection progresses (Figure 2d,e). A dry or water‐soaked to slimy‐brown rot of the roots and lower stalk may occur. On the ears of infected plants, bacterial ooze and freckles may also be observed in the inner husks. The abscission layer of kernels removed from infected ears during the soft dough stage may be slimy and discoloured orange (Jackson et al., 2007). *C. nebraskensis* is able to colonize and survive epiphytically on the surface of symptomless maize leaves. The bacterium has been detected in the phylloplane of maize seedlings 4 weeks after planting (Smidt & Vidaver, 1986a).

## 4 SIMILARITY TO OTHER DISEASES

Goss's wilt symptoms, development, and effect on yield are similar to Stewart's bacterial wilt and leaf blight caused by *Pantoea stewartii* subsp*. stewartii* (CABI, 2020; Pataky, 1989). In fact, the causal agent of Goss's wilt was once considered a more virulent and orange‐coloured strain of *P. stewartii* subsp*. stewartii* (Schuster, 1975). The morphological responses of a resistant maize hybrid to infection by *C. nebraskensis* were similar to those reported in maize inbred lines that were resistant to Stewart's wilt and may result from plant morphological characteristics such as plant height or genetic resistance (Mbofung et al., 2016; Pataky, 1985). Reactions to Goss's wilt and Stewart's wilt were highly correlated at 1% level of probability for 75 mid‐ to late‐season maturing sweetcorn hybrids evaluated in 1984 in an Illinois sweetcorn disease nursery (Pataky, 1985). The prevalence of Stewart's wilt during the 21st century has been almost negligible due to the widespread use of neonicotinoid seed treatments, which kill the flea beetle (*Chaetocnema pulicaria*) that vectors *P. stewartii* subsp*. stewartii* (Pataky et al., 2005). Goss's wilt may also be misdiagnosed as northern corn leaf blight (*Setosphaeria turcica*) or Diplodia leaf streak (*Stenocarpella macrospora*), which both cause large, elongated oval lesions on maize leaves (Malvick et al., 2018). In addition, nutrient deficiencies, for example of nitrogen, and leaf scorch caused by drought stress may be misdiagnosed as Goss's wilt.

## 5 ECONOMIC IMPACT OF THE DISEASE

The estimated total yield loss due to Goss's wilt from 2012 to 2015 was more than 1,270,000 tonnes in the United States and Canada, and Goss's wilt was among the top three most destructive maize diseases in the northern United States and Ontario (Ikley, 2019; Mueller et al., 2016). Losses decreased from 2016 to 2019, and the disease was ranked seventh, fifth, and ninth in 2016, 2017, and 2018, respectively, while it was not among the top 10 diseases in 2019 (Mueller et al., 2020). Increased availability of *C. nebraskensis‐*resistant hybrids is probably the reason for the decrease in yield losses. Individual field losses associated with the pathogen can be up to 60% based on disease severity, hybrid susceptibility, the growth stage at which the disease develops, and prevailing weather conditions (Carson & Wicks, 1991; Claflin, 1999; Malvick et al., 2014; Ruhl et al., 2009; Wise et al., 2010; Wysong et al., 1973). Correlation analyses suggest that for every 1% increase in R1 disease severity on a susceptible hybrid, yield is reduced by 117 kg/ha (1.9 bushels/acre) (Bauske & Friskop, 2021). In sweetcorn, yield decreased 17% and 19%, by weight and number of ears, respectively, for each 10% increase in severity (Pataky, 1989). No differences in yield loss were found among inoculation timings (six to 10 leaf collars [V6 to V10], reproductive silk stage [R1], or a sequential combination of V6 to V10 and R1) on a resistant hybrid. However, yield losses of 34%–41% and 22%–25% were observed on a susceptible hybrid and a moderately susceptible hybrid, respectively, when inoculation occurred at V6 to V10 (Bauske & Friskop, 2021). As far as grain quality is concerned, no differences in test weight or protein content in grain samples from the low disease plots compared to the high disease plots were detected, but there was a significant reduction in 1000‐seed weight in grain from the high disease plots (Robertson et al., 2012).

## 6 HOST RANGE OF THE PATHOGEN

In addition to maize, a number of gramineous plant species, that is, big bluestem (*Andropogon gerardii*), woolly cupgrass (*Eriochloa vollisa*), teosinte (*Euchlaena mexicana*), sugarcane (*Saccharum officinarum*), little bluestem (*Schizachyrium scoparium*), bristly foxtail (*Setaria verticillata*), giant foxtail (*Setaria faberi*), green foxtail (*Setaria viridis*), yellow foxtail (*Setaria pumila*), grain sorghum (*Sorghum vulgare*), shattercane (*Sorghum bicolor* subsp. *arundinaceum*), sudangrass (*S. bicolor* subsp. *drummondii*), and eastern gamagrass (*Tripsacum dactyloides*) are reported to act as secondary hosts for *C. nebraskensis* (Langemeier et al., 2012, 2014; Schuster, 1975; Webster et al., 2019). Furthermore, annual ryegrass (*Lolium multiflorum*), bristly foxtail, giant foxtail, green foxtail, yellow foxtail, Johnson grass (*Sorghum halepense*) and large crabgrass (*Digitaria sanguinalis*) have been reported to be hosts of *C. nebraskensis* based on symptom development in artificially inoculated plants (Ikley et al., 2015; Langemeier et al., 2014). The pathogen was isolated from barnyard grass (*Echinochloa crus‐galli*) although subsequent experiments failed to confirm that it could be infected (Ikley et al., 2015; Wysong et al., 1981). On annual ryegrass, giant foxtail, and Johnsongrass several days are required for initial lesion development, and lesion development occurred sooner when inoculum concentration increased from 100 to 10^7^ cfu/ml (Campbell et al., 2019). Neither systemic nor seedborne infection was demonstrated in these alternative hosts and the bacterium was restricted to inoculated leaf tissue (Ikley, 2019). Wheat and oats, both of which may be used as cover crops or in rotation with maize in the United States, were identified as potential hosts that maintained epiphytic or endophytic pathogen populations >10^6^ cfu/leaf sample, although there was no evidence of infection (Webster et al., 2019). Table 1 represents members of Poaceae plants infected or colonized under natural or experimental conditions by *C. nebraskensis*.

**TABLE 1:** Members of Poaceae plants infected or colonized under natural or experimental conditions by *Clavibacter nebraskensis*, the causal agent of Goss's wilt of maize

Species	Common name	Usage	Natural host	Experimental host	Epiphytic or endophytic	Systemic infection	Seedborne	Reference
*Zea mays*	Maize, corn	Crop	+	+	+	+	+	Schuster (1975); Webster et al. (2019); Langemeier et al. (2012, 2014); Ikley (2019)
*Andropogon gerardii*	Big bluestem	Forage	+	ND	ND	−	−	
*Setaria verticillata*	Bristly foxtail	Weed	+	+	ND	−	−	
*Tripsacum dactyloides*	Eastern gamagrass	Forage	+	ND	ND	−	−	
*Setaria faberi*	Giant foxtail	Weed	+	+	ND	−	−	
*Sorghum vulgare*	Grain sorghum	Crop	+		ND	−	−	
*Setaria viridis*	Green foxtail	Weed	+	+	ND	−	−	
*Schizachyrium scoparium*	Little bluestem	Ornamental	+	ND	ND	−	−	
*Sorghum bicolor* subsp*. arundinaceum*	Shattercane	Grass	+	ND	ND	−	−	
*S. bicolor* subsp*. drummondii*	Sudangrass	Grass	+	ND	ND	−	−	
*Saccharum officinarum*	Sugarcane	Crop	+	ND	ND	−	−	
*Euchlaena mexicana*	Teosinte	Fodder	+	ND	ND	−	−	
*Eriochloa villosa*	Woolly cupgrass	Weed	+	ND	ND	−	−	
*Setaria pumila*	Yellow foxtail	Weed	+	+	ND	−	−	
*Lolium multiflorum*	Annual ryegrass	Cover crop	ND	+	ND	−	−	Ikley et al. (2015); Langemeier et al. (2014)
*Sorghum halepense*	Johnson grass	Weed/forage	ND	+	ND	−	−	
*Digitaria sanguinalis*	Large crabgrass	Fodder	ND	+	ND	−	−	
*Echinochloa crus‐galli*	Barnyard grass	Fodder	+	−	ND	−	−	Wysong et al. (1981)
*Triticum aestivum*	Wheat	Crop	−	−	+	−	−	Webster et al. (2019)
*Avena sativa*	Oat	Crop	−	−	+	−	−	Webster et al. (2019)

*Note*: Except for maize, which is the main host of *C. nebraskensis*, the other plant species are considered either secondary or experimental hosts of the pathogen. +, positive; −, negative; ND, not determined.

*C. nebraskensis* survives epiphytically on symptomless maize leaves and it is possible that populations build up gradually over time in new areas before disease is detected (Eggenberger et al., 2016). The population of the pathogen in residue is highest just after harvest and declines by four or five orders of magnitude over the winter and summer (Smidt & Vidaver, 1986a, 1986b). Infection is favoured by warm weather (26–32°C) (Smidt & Vidaver, 1986a) and high relative humidity (Mallowa et al., 2016). Warm and dry conditions can limit the development of Goss's wilt and leaf blight (Jackson et al., 2007). In general, more susceptible maize hybrids maintained larger populations of *C. nebraskensis*, but occasionally large populations are found on intermediate and tolerant hybrids (Schuster et al., 1983). The wilt phase of the disease is less common and usually occurs on young plants or severely blighted plants, while the leaf blight phase may occur at any stage of growth (Mbofung et al., 2016; Ruhl et al., 2009).

Epiphytic populations of *C. nebraskensis* on host and nonhost plant species under favourable conditions could be an important source of inoculum for the establishment of the pathogen in the upcoming season. Epiphytic *C. nebraskensis* was detected on symptomless maize leaves collected up to 2.5 m away from inoculum sources 15 days after inoculation in the field assays (Eggenberger et al., 2016). Furthermore, other coryneform plant pathogens, for example *C. flaccumfaciens* pv. *flaccumfaciens*, can accompany *C. nebraskensis* in the maize phyllosphere, interfering with accurate isolation and identification of the Goss's wilt pathogen (Figure 4c,d; Harveson et al., 2015; Osdaghi et al., 2020b). Ten grass species, including wheat and oats, have been identified as potential sustaining hosts that maintain epiphytic or endophytic pathogen populations >10^6^ cfu/leaf (Campbell et al., 2019; Webster et al., 2019).

## 7 BACTERIOLOGICAL FEATURES OF THE PATHOGEN

*C. nebraskensis* is a gram‐positive, orange‐pigmented, nonacid‐fast, nonspore‐forming, nonmotile (without flagella), pleomorphic, rod‐shaped bacterium. Club‐shaped, ellipsoidal, ovoid, or rarely “whip‐handle” cells may also be observed. The cells are approximately 0.5 × 1–2.0 μm, often grouped in angular and palisade arrangements (Bradbury, 1991; Vidaver & Mandel, 1974). Growth on nutrient broth yeast extract agar, potato dextrose agar, and a synthetic medium supplemented with yeast extract (necessary for growth) is slow, with visible colonies rarely appearing before 3–4 days have passed (Figure 4a). The bacterium produces four morphological colony types: orange fluidal colonies typical of the species (Figure 4b), a nonfluidal dark‐orange variant, a fluidal yellow variant, and a fluidal slightly orange‐pigmented variant that in younger colonies appears almost white at room temperature (22–26°C) (Saddler & Kerr, 2015; Smidt & Vidaver, 1987). The intensity of pigmentation, however, varies with the temperature and medium (Vidaver & Mandel, 1974). *C. nebraskensis* may be characterized by its inability to grow on 0.005% triphenyltetrazolium chloride agar (Vidaver & Mandel, 1974). The optimum temperature for *C. nebraskensis* growth in vivo is 27°C while the bacterium can survive between 12 and 38°C (Smidt & Vidaver, 1986b). Loss of virulence has been reported on culture medium and can be prevented by storage in appropriate conditions, for example 15% sterile glycerol at −80°C (Schuster et al., 1975). Storage of the bacterial cells in sterile distilled or deionized water, or phosphate buffer at 6°C or room temperature (24 ± 3°C) will give rise to colony type variants and is unsatisfactory for maintenance of viability and virulence (Vidaver, 1977). Viability and virulence of *C. nebraskensis* are optimal either on solid complex media maintained at 6°C for 2 years or as lyophilized cultures maintained at −20°C for up to 5 years. Bacterial strains maintained in dried leaves of greenhouse‐grown plants are viable and virulent for up to 3 months (Vidaver, 1977).

## 8 GENETIC DIVERSITY AND POPULATION STRUCTURE

Bacteriocin profile, colony morphology, and pigmentation are among the most variable phenotypic features of plant‐pathogenic coryneform bacteria (Chen et al., 2021; Hamidizade et al., 2020; Osdaghi et al., 2016, 2018b, 2018c; Vidaver, 1982). No variation in the population of *C. nebraskensis* was observed during the first decade of its description (Vidaver et al., 1981). However, morphological differences were observed in *C. nebraskensis* strains isolated from one popcorn field in 1982 (Smidt & Vidaver, 1987). Bacteriophage and bacteriocin screening typed 85 *C. nebraskensis* strains collected between 1969 and 1979 into eight groups (Shirako & Vidaver, 1981; Vidaver et al., 1981). No correlation was found between groups and either the year of isolation or the geographic origin of the strain. Suparyono (1989) grouped 50 *C. nebraskensis* strains isolated in Nebraska into seven major groups based on colony colour and morphology on solid media and bacteriocin production. The strains were further divided by bacteriophage sensitivity.

Although several comprehensive investigations estimated the genetic diversity and population structure of other coryneform plant‐pathogenic bacteria (Ansari et al., 2019; Jacques et al., 2012; Osdaghi et al., 2022a), until recently there were few reports regarding the genetic diversity and phylogeny of *C. nebraskensis*. Repetitive sequence‐derived (rep)‐PCR profiling using the BOX‐A1R primer showed *C. nebraskensis* is closely related to the tomato pathogen *C. michiganensis* and distinct from the other species of the genus *Clavibacter* (Smith et al., 2001). Using amplified fragment length polymorphism (AFLP) analysis and repetitive DNA sequence‐based BOX‐PCR, Agarkova et al. (2011) reported 131 *C. nebraskensis* strains collected between 1969 and 2009 clustered into two groups, A and B, where group B represents recent genetic changes between 1999 and 2009 while the genome of group A had been stable for a long period of time. Similarly, Langemeier et al. (2012) used AFLP and rep‐PCR DNA to separate 466 strains collected in the United States into three main groups. No geographical basis for groupings was noted. *C. nebraskensis* strains isolated from symptomatic and asymptomatic maize leaves varied in aggressiveness, in terms of proportion of leaf area infected (Ahmad et al., 2015). However, while sequence polymorphisms were detected in five putative virulence genes of *C. nebraskensis*, that is, cellulase A (*celA*), two endoglucanases, xylanase B, and a pectate lyase, there was no relationship with pathogenicity (Ahmad et al., 2015). Using multilocus sequence analysis and typing (MLSA/MLST), Webster et al. (2020) suggested that genetic changes in the population of *C. nebraskensis* were correlated with recent geographic expansions of Goss's wilt outbreaks in the north‐central United States. Moreover, a significant substructure was detected between subpopulations from historic outbreaks in Nebraska and Colorado and more recent outbreaks in Minnesota. Webster et al. (2020) noted that 34% of the *C. nebraskensis* strains evaluated were classified as either weakly virulent or very weak virulence/avirulent, while virulence or avirulence in maize was not correlated with a particular MLSA scheme.

## 9 GENOMIC FEATURES OF THE PATHOGEN

The first draft genome sequence of *C. nebraskensis* (DOAB 397, isolated in Manitoba, Canada) became available in 2015 (Tambong et al., 2015). However, the total number of publicly available genome resources of the species is limited. In March 2022, fewer than 10 whole genome resources of *C. nebraskensis* were available in the NCBI GenBank database (Figure 1). The chromosome of *C. nebraskensis* is mostly collinear, like the tomato pathogen *C. michiganensis*, and both species are free of insertion elements (Eichenlaub & Gartemann, 2011). Comparative genomics analysis showed *C. nebraskensis* has the smallest genome among all species in the genus *Clavibacter*, and the fewest protein‐coding genes, while the species does not display a reduced genome like other genome‐reduced coryneform species, for example the sugarcane pathogen *Leifsonia xyli* subsp. *xyli* (Monteiro‐Vitorello et al., 2004; Tambong, 2017; Zaluga et al., 2014). The proteomes of *C. nebraskensis* strains DOAB 395 and DOAB 397 had 99.2% homology, while they had 92.1% and 91.8% homology, respectively, with the strain NCPPB 2581^T^ (Tambong et al., 2016). *C. sepedonicus* and *C. nebraskensis* possess similar sets of extracellular enzymes to each other, although some of the genes are not orthologous and both species lack genes orthologous to *tomA* in *C. michiganensis* (Eichenlaub & Gartemann, 2011). Interestingly, no related proteases of the Chp or Ppa families have been identified in the genome of *C. nebraskensis*, suggesting a different virulence mechanism is operative in monocotyledonous plants. The only protein suspected to be involved in the pathogenicity of *C. nebraskensis* is an anion channel‐forming protein (Eichenlaub & Gartemann, 2011; Michalke et al., 2001; Schürholz et al., 1991). Southern blot hybridization data showed that *C. insidiosus*, *C. nebraskensis*, and *C. tessellarius* contain sequences hybridizing to the cellulase gene from *C. michiganensis*, but *Rathayibacter iranicus*, *R. tritici*, and *R. toxicus*, once classified within the genus *Clavibacter* (Riley & Ophel, 1992), do not (Dreier et al., 1995).

Extrachromosomal plasmids were reported in *C. insidiosus*, *C. michiganensis*, *C. sepedonicus*, and several *C. nebraskensis* strains (Gross et al., 1979; Gross & Vidaver, 1979; Vidaver, 1982). However, the genome of the type strain of the Goss's wilt pathogen NCPPB 2581^T^ is not known to carry plasmids (Eichenlaub & Gartemann, 2011). The chromosome of NCPPB 2581^T^ contains protein families with homologies in at least one of the plasmids of *C. michiganensis*, *C. sepedonicus*, *C. capsici*, and *C. insidiosus* (Tambong, 2017). Plasmids are not required for the pathogenicity of *C. nebraskensis* because most maize‐pathogenic strains do not carry a plasmid (Eichenlaub & Gartemann, 2011; Tambong, 2017). Thus, it can be hypothesized that the disease‐inducing virulence factors are chromosomally encoded alongside genes involved in successful host colonization. Furthermore, 28 plasmid‐borne coding sequences (CDSs) in the other *Clavibacter* species were found to have homologues in the chromosomal genome of *C. nebraskensis* NCPPB 2581^T^. These CDSs include pathogenesis‐related factors such as endocellulases E1 and β‐glucosidase (Tambong, 2017).

## 10 PATHOGENICITY MECHANISMS

Among the economically important gram‐positive bacterial plant pathogens, *C. nebraskensis* is one of the least studied members in terms of virulence repertoires and pathogenicity determinates (Hwang et al., 2018, 2020; Laine et al., 1996; Lu et al., 2015; Mogen & Oleson, 1987; Nissinen et al., 2009; Osdaghi et al., 2018d; Thapa et al., 2019). Progress in determining the pathogenicity mechanisms of *C. nebraskensis* has lagged behind that for the tomato and potato pathogens *C. michiganensis* and *C. sepedonicus*, respectively (Eichenlaub & Gartemann, 2011; Osdaghi et al., 2022b). The reason for this may be that genetic manipulation technologies and transformation vectors have not yet been developed for the maize pathogen, although it is possible that platforms for the closely related pathogens, for example *C. michiganensis* and *C. sepedonicus*, could be effective (Thapa et al., 2017). For instance, the vector pDM100, which has been used for transformation of *C. michiganensis*, has also been reported to transform *C. nebraskensis* and *C. insidiosus* but at lower rates (Meletzus & Eichenlaub, 1991). Recently, Mullens and Jamann (2021) successfully introduced the green fluorescent protein (GFP)‐expressing plasmid pK2‐22 (Chalupowicz et al., 2012) into *C. nebraskensis* and used these labelled bacteria to monitor the bacteria in planta. This indicates that pCM1‐based plasmids can be introduced and stably maintained in *C. nebraskensis*.

While gram‐negative plant‐pathogenic bacterial pathogens translocate a cocktail of different effector proteins (referred to as type III effectors) into host plant cells using the type III secretion system (TTSS) (Shah et al., 2021), the TTSS is absent from gram‐positive bacteria. Instead, the latter group possesses a series of lytic enzymes and toxic compounds, and in some cases relies on extrachromosomal plasmids to initiate virulence on their host plant (Chen et al., 2021; Eichenlaub & Gartemann, 2011; Francis et al., 2010; Hogenhout & Loria, 2008; Thapa et al., 2019). Most of the members of *Clavibacter* spp. rely on exopolysaccharides and enzymes such as endocellulase, xylanase, polygalacturonase, and serine protease as major candidate contributors to pathogenicity (Bentley et al., 2008; Stevens et al., 2021; Thapa et al., 2017, 2019). Members of the genus *Clavibacter* also produce phytotoxins, which are high molecular mass polysaccharides (Rai & Strobel, 1969; Van Alfen & McMillan, 1982). In *C. nebraskensis* culture supernatants a 65 kDa protein was identified as an active moiety, forming anion channels in planar lipid bilayers (Schürholz et al., 1991). This protein may be unique to this species because the activity is not found in culture supernatants from *C. michiganensis*. The formation of membrane channels is a common mode of action for toxins, thus this protein could be a toxin involved in pathogenesis (Metzler et al., 1997; Schürholz et al., 1991). Recently, Owusu et al. (2019) provided novel insights to the role of phytoglobins (Pgbs) during the maize and *C. nebraskensis* interactions. Suppression of ZmPgb1.1 is effective in reducing lesion size and disease severity symptoms in maize leaves infected with the pathogen.

Strains of *C. nebraskensis* vary in aggressiveness on maize (Soliman et al., 2021), and virulence or avirulence in maize is not correlated with a particular MLSA‐based phylogeny (Webster et al., 2020). Among 33 candidate virulence genes, sequence polymorphisms were found in only five genes: cellulase A, two endoglucanases, xylanase B, and a pectate lyase (Ahmad et al., 2015). However, no relationship was found between the polymorphisms present and the pathogenicity of the strains. Wilting is frequently observed when younger plants are infected. The pathogen deploys virulence factors, for example cell wall‐degrading enzymes and proteases, that affect the integrity of the host plant's cell walls and membranes (Mbofung et al., 2016; Zaluga et al., 2014). Considering the differences in cell wall compositions between *Clavibacter* plant hosts, it is probable that some of these cell wall‐degrading enzymes could be required for the colonization of maize specifically. Differential secretomes have been detected in the presence of different xylem sap compositions, for example maize versus tomato. Virulence factors in xylem sap, for example cellulase, β‐glucosidase, β‐galactosidase, chitinase, β‐1,4‐xylanase, and proteases, were generally more abundant in aggressive strains. These proteins were either not detected or detected at significantly lower abundance levels in nonhost xylem sap (tomato), suggesting they are host‐specific factors involved in *C. nebraskensis–*maize interactions (Soliman et al., 2021). It is therefore possible that the expression and presence of some virulence factors may be a result of a host‐dependent interaction.

Among the plant‐pathogenic members of *Clavibacter*, putative genes associated with pathogenicity may carry different roles, which may depend on their plant host. In *C. michiganensis*, some *chp* serine proteases are known to not affect virulence when deleted (Peritore‐Galve et al., 2021) and some are recognized to restrict pathogen growth and disease in related plant species outside of tomato (Park et al., 2022; Verma & Teper, 2022). Conversely, in *C. capsici*, one *chp* protease (*chpG*) is a putative pathogenicity factor critical for disease as knockouts and natural variants lacking *chpG* are less virulent (Hwang et al., 2020). Ahmad et al. (2015) found many putative secreted serine proteases in two strains of *C. nebraskensis* while their involvement could be related to colonization and host resource utilization. Homologues of secreted serine protease of the Chp/Pat‐1 and Ppa families have not yet been reported in *C. nebraskensis*, which is surprising due to the key role of these compounds in the virulence and host specificity of other *Clavibacter* speci, for example *C. michiganensis*, *C. sepedonicus*, and *C. capsici* (Park et al., 2022).

## 11 BIOLOGY AND EPIDEMIOLOGY OF THE PATHOGEN

The main predictors of Goss's wilt incidence in a given area where the pathogen has been reported are hybrid resistance, planting population density (>67,500 plants/ha), longitude, planting date, crop rotation, percentage of residue, yield history, tillage, and growth stage (Jardine & Claflin, 2016; Langemeier, 2012; Langemeier et al., 2017). The primary inoculum source of *C. nebraskensis* is infested crop residue on the soil surface (Schuster, 1975; Figure 2i). Infections predominantly occur through wounds on the maize plant created by heavy rain, hail, or sand blasting during rainstorms. The earlier that seedling infection occurs, the higher the disease severity is, while inoculation timing has very little impact in a resistant hybrid (Calub et al., 1974; Suparyono, 1989). Disease severity was significantly reduced when roots were inoculated compared to both leaf and stem inoculations, which were not significantly different from each other (Mehl et al., 2021). In resistant hybrids, the lesions are smaller and the leaf tissue surrounding the lesion is often reddish (Mbofung et al., 2016). The pathogen survives over winter in all plant parts and has been isolated from most plant parts, including roots and stems, leaf blades and sheaths, tassels, husks, silks, cobs, and kernels (Gross & Vidaver, 1979; Schuster, 1975; Smidt & Vidaver, 1986a). Wind can deposit infected debris in nearby fields or transfer epiphytic populations of the bacterium from leaf to leaf as leaves rub together in the wind (Graham & Harrison, 1975; Venette & Kennedy, 1975). Frequently, the onset of infection is seen following severe summer storms during which wind and water droplets can disseminate inoculum within and between maize fields (Jackson et al., 2007). Injury from wind or sand provides avenues for infection (Mallowa et al., 2016; Rocheford et al., 1985b). However, the bacterium can multiply on noninjured maize leaves and initiate infections via hydathodes, the base of trichomes, and stomata (Mallowa et al., 2016; Mullens & Jamann, 2021).

**FIGURE 3:**
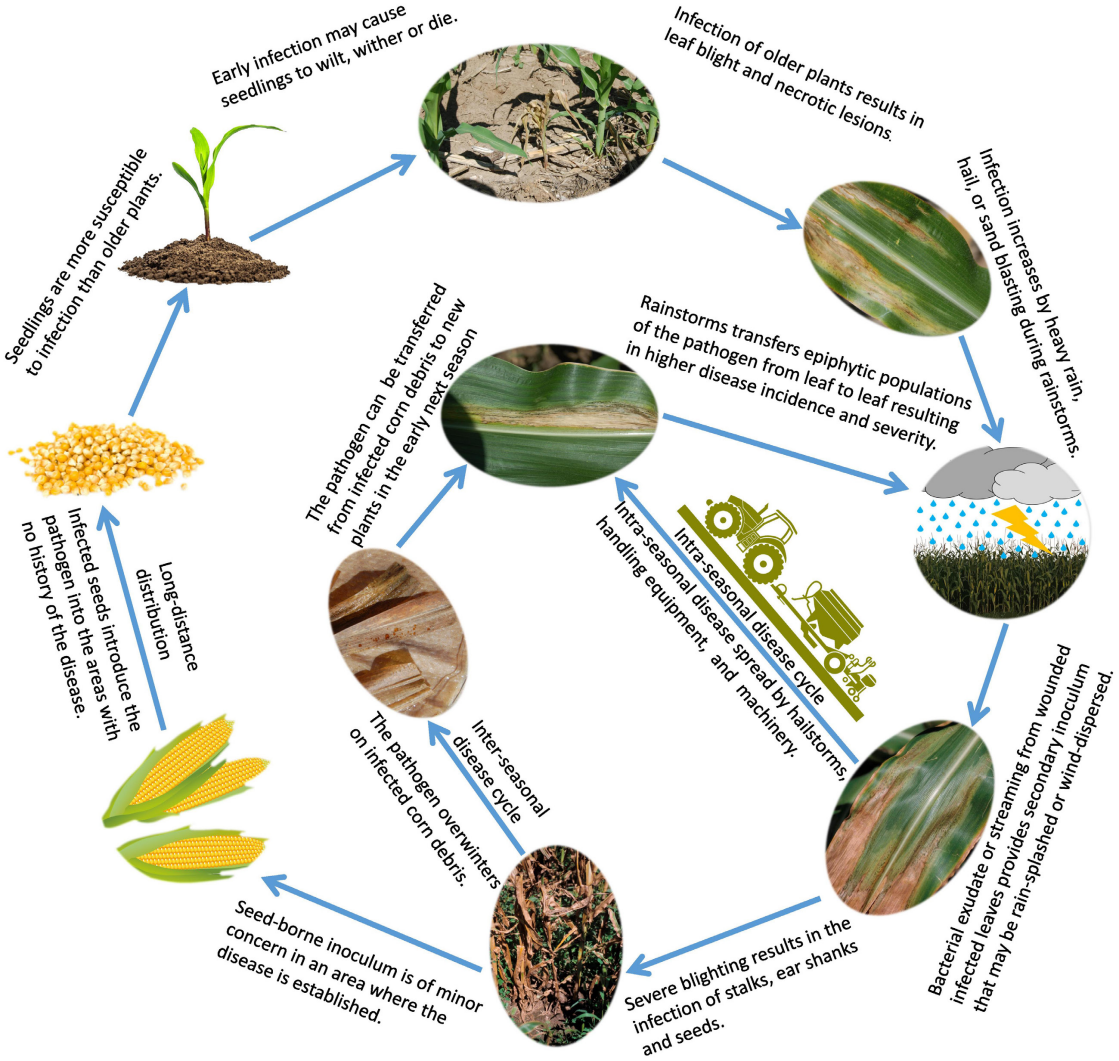
Disease cycle of Goss's wilt of maize caused by *Clavibacter nebraskensis*

Leaf surface waxes affect leaf colonization by *C. nebraskensis*; lower density crystalline waxes favour bacterial colonization (Beattie & Marcell, 2002). The pathogen primarily infects the host plant through wounds caused by wind, farm machinery, hailstorms, or wind‐blown sand and insect feeding (Jackson et al., 2007; Owusu et al., 2019). However, wounding is not required for infection as previously thought. Stomata, hydathodes, and the base of trichomes provide entry points for the bacterium as well (Mallowa et al., 2016; Mullens & Jamann, 2021). On infection, *C. nebraskensis* enters the xylem tissue (Caldwell & Iyer‐Pascuzzi, 2019; Mullens & Jamann, 2021) and spreads bidirectionally (Mullens & Jamann, 2021). Degradation of the xylem walls by the bacterium enables it to spread and colonize the mesophyll (Mullens & Jamann, 2021). Mullens and Jamann (2021) clearly showed the bacteria move unidirectionally from the xylem to the mesophyll, but were not capable of moving from the mesophyll to the xylem. Bacterial exudate or streaming from wounded infected leaves provides secondary inoculum that may be rain‐splashed or wind‐dispersed. Figure 3 illustrates the disease cycle of Goss's wilt under natural field conditions.

## 12 SEED TRANSMISSION

*C. nebraskensis* has been detected in seeds, both externally and internally, and may be found in the vicinity of the endosperm, scutellum, and embryo (Biddle et al., 1990; Schuster, 1975). Superficial contamination of the maize seed coat occurs routinely under natural conditions in a rate of 20.8%–32.0% (Jackson et al., 2007; Schuster, 1975). The pathogen was detected in the seeds, ear shanks, and stalks of susceptible maize after leaves were inoculated in the field. The percentage of seeds infected ranged from 17.1% to 30.7% (Biddle et al., 1990). While seed transmission of *C. nebraskensis* has been demonstrated, it is generally uncommon and has been a matter of conflict among scientists. Transmission of the pathogen from the mother plant to seeds was demonstrated by recovery of the bacterium from stems, shanks, and seeds (externally and internally) of inoculated plants (Biddle et al., 1990). Severe blighting of the ear leaf corresponded to greater numbers of the bacterium detected on both the exterior and interior of seed collected from inoculated plants. Internal populations of the pathogen and the percentage of infected seeds were not affected by either moisture content at harvest (38% or 25%) or drying at 35°C (Biddle et al., 1990).

Seed transmission has not been observed in naturally infected seeds in greenhouse grow‐outs and field planting; however, in seeds that were vacuum‐infiltrated with *C. nebraskensis*, transmission was observed in greenhouse plantings at rates of 0.1%–0.4% (Biddle et al., 1990). Shepherd (1999) reported a seed transmission rate of 0.04% (one of 2568 plants) in a greenhouse planting from a naturally infected seed lot. More recently, Block et al. (2019) reported 12 seed transmission events among 241,850 plants grown from seed lots harvested from a field trial in which Goss's leaf blight severity ranged from 3.6% to 37.0%; a seed transmission rate of 0.005% for the pathogen, or 0.04% when the seed transmission rate was calculated using only infected seeds. Hence, it seems unlikely that seed transmission could introduce enough inoculum to create a serious disease outbreak in a single growing season. Based on these data, seedborne inoculum is of minor concern in an area where the disease is established, although infected seeds could introduce the pathogen into the areas with no history of the disease (Biddle et al., 1990). Indeed, Eggenberger et al. (2016) suggested the Goss's wilt outbreaks during the mid‐2000s were more probably due to the gradual, undetected build‐up of inoculum on crop residue from susceptible hybrids grown in a continuous maize system rather than dispersal from point sources such as a seed transmission event in a single season. However, even this trace percentage of seed transmission is relevant for phytosanitary restrictions and preventing the introduction of the pathogen to new areas (Block et al., 2019). Shepherd et al. (2017) detailed a seed health testing method for the detection of *C. nebraskensis* (USDA National Seed Health System Standard Method, www.seedhealth.org) that is based on the original method described by Shepherd (1999).

## 13 ISOLATION, DETECTION, AND IDENTIFICATION OF THE PATHOGEN

Quarantine regulations in many countries are in place to prevent the introduction of *C. nebraskensis* through commercial maize seed (Carson & Wicks, 1991). Traditionally, confirmation of *C. nebraskensis* from various tissues includes observation of symptoms, culture plating, and inoculation of indicator plants. These techniques are used because of their simplicity, but they are time‐consuming (Singh & Somerville, 1992).

**FIGURE 4:**
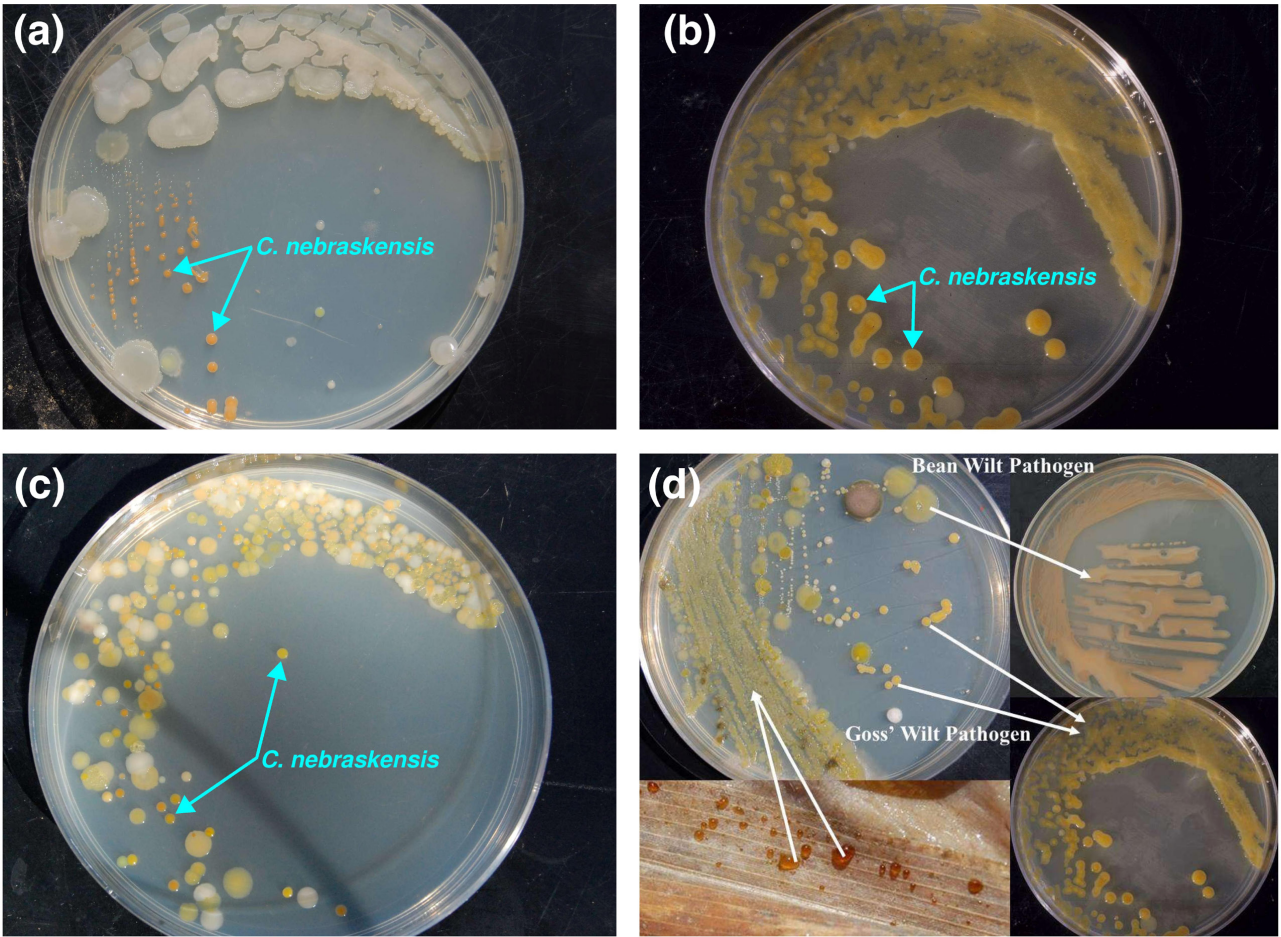
Colony characteristics of *Clavibacter nebraskensis* strains isolated from Goss's wilt symptoms of maize tissues on nutrient broth‐yeast extract (NBY) medium. Growth on NBY medium is slow, with visible colonies rarely appearing before 3–4 days (a). The bacterium produces orange fluidal colonies typical of the species on NBY (b). Presence of other coryneform plant pathogens, for example *Curtobacterium flaccumfaciens* pv. *flaccumfaciens*, can accompany *C. nebraskensis* in the maize phyllosphere, interfering with accurate isolation and identification of the Goss's wilt pathogen (c,d)

Similar to the other coryneform phytopathogenic bacteria, *C. nebraskensis* can be isolated on enriched media, for example yeast extract peptone glucose agar (YPGA: yeast extract 5 g, peptone 5 g, glucose 10 g, agar 15 g in 1 L distilled water) and nutrient broth‐yeast extract (NBY: nutrient broth 8 g, yeast extract 2 g, K_2_HPO_4_ 2 g, KH_2_PO_4_ 0.5 g, MgSO_4_ 0.25 g, glucose 5 g, agar 15 g in 1 L distilled water) media. Gross and Vidaver (1979) developed a selective agar medium (CNS) for isolating *C. nebraskensis* from maize tissue and soil. Colonies of *C. nebraskensis* are yellow or apricot‐orange, round, convex, glistening, butyrous, and entire, 4 mm in diameter after 6 days growth at 25°C on YPGA, NBY or CNS media (Figure 4a–d; Gross & Vidaver, 1979). On potato dextrose agar (PDA) medium colonies are white to cream, but the addition of thiamine increases growth and promotes the development of an orange pigment (Bradbury, 1991). Another selective medium (NSM) for isolating *C. nebraskensis* has been developed in Japan (Aizawa et al., 1997). On NSM, *C. nebraskensis* forms capitated, smooth and salmon‐pink colonies after 3–4 days of incubation at 30°C and blackening of the medium around the colonies is observed. Using the latter colony morphology and colour, the pathogen can easily be distinguished from other coryneform phytopathogenic bacteria such as *C. michiganensis*, *C. flaccumfaciens* pv. *flaccumfaciens*, and *Rhodococus fascians*, which can grow on NSM.

The GEN III OmniLog identification system (Biolog Inc.) provides accurate identification of *Clavibacter* species. Similarly, serological techniques such as ELISA are available but not routinely used for identification of *C. nebraskensis,* mainly due to the lack of species specificity (Korus, 2011; Louws et al., 1998). A novel method based on microsphere immunoreaction with fluorescent labels such as quantum dots and R‐phycoerythrin has been developed for the simultaneous detection of *C. nebraskensis* and *Pseudomonas syringae* pv. *syringae* in maize (Zhang et al., 2013). The limit of detection for the latter method is 10 times lower than ELISA, and its analysis time (1 h) is much shorter compared to ELISA (6–8 h) (Zhang et al., 2013).

There are several high‐throughput molecular techniques also available for detection and identification of *C. nebraskensis*. A species‐specific PCR assay was developed for the five species of *Clavibacter* where the size of the amplified specific DNA fragment for *C. nebraskensis* using a primer pair (PSA‐5) is 393 bp (Pastrik & Rainey, 1997a). Using primer pair PSM1 and CM3, the pathogen can be detected in infected maize seed and seedlings by PCR amplification of a 215 bp DNA fragment (Ayala‐Labarrios et al., 2004). RAPD‐PCR has been used to differentiate strains of different species within the genus *Clavibacter* (Pastrik & Rainey, 1997a, 1997b). A nested‐PCR method based on internal transcribed spacer (ITS) sequence using the primers CmnFP‐OUTER/CmnRP‐OUTER and CmnFP‐rNNER/CmnRP‐INNER results in amplification of a 122 bp DNA fragment from *C. nebraskensis* strains but not other *Clavibacter* species (Feng et al., 2004). This nested‐PCR detection method can detect *C. nebraskensis* with a minimum limit of 354 cfu/ml, and the sensitivity of the method is 1000 times higher than that of the conventional PCR method (Feng et al., 2004, 2014). Bach et al. (2003) developed a real‐time TaqMan PCR assay to detect and quantify pathogenic *Clavibacter* species, including *C. nebraskensis*, in infected tissues. Tambong et al. (2016) developed a more specific multiplex TaqMan real‐time PCR tool for rapid detection of *C. nebraskensis* where its sensitivity (0.1–1 pg) compared favourably with previously reported real‐time PCR assays. Using the CMN_01184 gene, McNally et al. (2016) developed conventional PCR and SYBR Green‐based quantitative PCR (qPCR) assays for specific detection and quantification of *C. nebraskensis*. Detection limits were determined at 30 and 3 ng of pure *C. nebraskensis* DNA, and 100 and 10 cfu of *C. nebraskensis* for the conventional PCR and qPCR assays, respectively. Recently, a robust and rapid multiplex TaqMan qPCR was developed by Larrea‐Sarmiento et al. (2019) to detect members of *Clavibacter* in general and *C. nebraskensis* with enhanced reliability and accuracy by adding noncomplementary AT sequences to the 5′ end of the forward and reverse primers. The assay is capable of detecting from 100 fg of *C. nebraskensis* genome targets. A number of PCR‐based detection methods are commercialized in the market (https://orders.agdia.com/amplifyrp‐xrt‐for‐cmn‐xcs‐70100).

Xu et al. (2021) developed and tested an amplicon‐based Nanopore detection system for *C. nebraskensis*, targeting a purine permease gene. The sensitivity (1 pg) of this system in mock bacterial communities spiked with serially diluted DNA of *C. nebraskensis* NCPPB 2581^T^ is comparable to that of real‐time qPCR. A loop‐mediated amplification (LAMP) assay is available for specific detection of *C. nebraskensis* under field conditions (Yasuhara‐Bell et al., 2016). The LAMP assay is run in a hand‐held real‐time monitoring device (SMART‐DART) and performs as well as in‐laboratory quantitative PCR equipment. Dobhal et al. (2019) implemented the whole‐genome comparative genomics approach to identify a unique and conserved region within all known species of *Clavibacter* and developed a sensitive, specific, and robust LAMP assay for detection of all known *Clavibacter* species, including *C. nebraskensis*.

## 14 MANAGEMENT

Goss's wilt monitoring and subsequent management should be priorities in areas where the disease has been reported (Harding et al., 2018). Planting pathogen‐free seed in areas with no history of the disease may prevent the introduction of the pathogen. Given the importance of maize crop residues as an inoculum source for the pathogen (Schuster, 1975; Smidt & Vidaver, 1986a), crop rotation and field sanitary practices, for example destruction of maize debris, were recommended for successful management of Goss's wilt (Wysong et al., 1981). However, this is rarely recommended now because crop residues left on the surface provide numerous environmental advantages, that is, reduced erosion, improved water infiltration, cooler soil temperatures, and increased soil organic matter, that outweigh concerns about increased inoculum (Blanco‐Canqui & Lal, 2009; Wilhelm et al., 2007). In 2016, two‐thirds of the maize hectarage in North America was grown under conservation tillage. Good weed management can reduce the risk of Goss's wilt because many weeds are alternative hosts of the bacterium (Jackson et al., 2007). Bacteriophages have been described for management of *C. nebraskensis* (Shirako et al., 1986; Smidt & Vidaver, 1987) although they are not widely used. Seed coating of maize with CN8 bacteriophage reduced the bacterial load by up to 76% (Kimmelshue et al., 2019). Polyvinyl alcohol‐stabilized CN8 bacteriophages on seed when coatings did not contain a stabilizer and, when combined with whey protein, CN8 bacteriophage activity was maintained in storage for 4 months at 26°C and 7 months at 10°C (Kimmelshue et al., 2019).

Currently there is no specific chemical control method for Goss's wilt in the field (Jackson et al., 2007; Lamichhane et al., 2018; Sisson et al., 2016). However, Goss's wilt control could be partially achieved as a side effect of controlling other biotic constraints. For instance, application of foliar fungicides on maize is useful for management of grey leaf spot (*Cercospora zeae‐maydis*) and northern corn leaf blight (*S. turcica*) as well as Goss's wilt (Obura, 2015). Using herbicides to control *C. nebraskensis*‐infected weeds did not reduce the pathogenicity of the bacterium recovered from treated plants (Ikley, 2019). Indeed, very few options for managing Goss' wilt other than genetic resistance are available for growers. However, it could be difficult for some producers to have access to hybrids designed for their region with that trait. This process takes time and other methods may have to be developed until that time occurs. Copper‐based products have been shown, in some instances, to increase economic returns and reduce losses due to certain bacterial diseases in dry beans, such as halo blight, caused by *Pseudomonas savastanoi* pv*. phaseolicola* and brown spot, caused by *P. syringae* pv. *syringae*, but not consistently for others (Garrett & Schwartz, 1998; Legard & Schwartz, 1987). However, several new copper‐alternative chemicals have recently been demonstrated to effectively manage a complex of multiple bacterial diseases in dry beans by producing higher yields without reducing disease incidence (Harveson, 2019). To our knowledge, none of these products has been tested on Goss's wilt in maize and this could be another direction to follow in efforts to identify and construct new methods to manage this disease.

## 15 HOST RESISTANCE

The use of tolerant and resistant hybrids is recommended as the best means for managing Goss's wilt. Indeed, the decreased prevalence of Goss's wilt in the United States after 2016 is mostly due to the use of *C. nebraskensis*‐resistant hybrids. While no maize genotype is completely immune to the pathogen (Mullens, 2020), various levels of resistance occur in maize lines (Biddle et al., 1990; Block et al., 2019; Calub et al., 1974; Ngong‐Nassah et al., 1992; Schuster, 1975; Soliman et al., 2018; Treat & Tracy, 1990; Wysong et al., 1981). Resistance is quantitatively inherited (Rocheford et al., 1985). Recently, Mehl et al. (2021) screened over 1000 inbred maize lines from the University of Illinois maize inbred collection and reported 21 lines belonging to the Lancaster heterotic family that showed acceptable levels of resistance against *C. nebraskensis*. Association mapping identified major quantitative trait loci (QTLs) for resistance to Goss's wilt (Schaefer & Bernardo, 2013; Singh et al., 2016). Schaefer and Bernardo (2013) identified nine QTLs for resistance to Goss's wilt, while 11 QTLs were detected for resistance to Goss's wilt on chromosomes 1, 2, 3, 4, 5, and 10 through joint linkage mapping (Singh et al., 2016). Most recently, Cooper et al. (2018) identified a QTL on chromosome 1, designated qGW1.06, which is a strong candidate for further characterization of resistance against Goss's wilt.

Resistance to *C. nebraskensis* involves both vascular and nonvascular components. Resistant maize lines exhibited decreased bacterial spread in the vasculature and the mesophyll (Mullens, 2020). Mbofung et al. (2016) observed that resistance to *C. nebraskensis* was associated with production of a dense matrix in the xylem that deformed and restricted movement of the bacterial cells. Wound inoculation of susceptible and resistant maize hybrids at the V4 to V5 developmental stage revealed that the pathogen multiplied and spread within the tissues of susceptible lines at a faster rate in comparison to a resistant hybrid (Mbofung et al., 2016). The pathogen colonized the metaxylem vessels in both susceptible and resistant maize hybrids (Mbofung et al., 2016; Mullens & Jamann, 2021). Spread from cell to cell was accomplished through disruption of cell walls, presumably from abundance of bacterial cells or enzymatic activity (Mbofung et al., 2016). Localized cell death similar to a hypersensitive response and vascular defence responses that resulted in the suppression of bacterial growth in maize veins have been reported (Mullens, 2020).

Phytoglobins (Pgbs) levels in maize plants influenced the resistance against *C. nebraskensis* through NO‐, ethylene‐, reactive oxygen species (ROS)‐, and programmed cell death (PCD)‐related defence mechanisms. Suppression of the maize phytoglobin, ZmPgb1.1, reduced lesion size and disease severity in leaves following artificial inoculation with the pathogen (Owusu et al., 2019). Resistance to Goss's wilt correlates positively with resistance to a number of maize diseases. For instance, reactions to Goss's wilt and Stewart's bacterial wilt (caused by *P. stewartii* subsp*. stewartii*) are highly correlated (Pataky, 1985; Pataky et al., 2011). Resistance to Goss's wilt is correlated with resistance to northern leaf blight (*S. turcica*). However, Goss's wilt resistance does not correlate with resistance to grey leaf spot (*Cercospora* spp.) nor southern leaf blight (*Cochliobolus heterostrophus*) (Cooper et al., 2018). Moreover, resistance to Goss's wilt is associated with common rust resistance (Hu et al., 2018). On the other hand, Goss's wilt‐resistant maize plants, when pretreated with bacteria, became more vulnerable to the subsequent attack by the fall armyworm (*Spodoptera frugiperda*) larvae (Da Silva et al., 2021).

## 16 CONCLUSION AND FUTURE AVENUES FOR RESEARCH

To date, Goss's wilt has not been detected outside North America. While the risk of seed transmission is very low, the risk is not zero (Block et al., 2019). Goss's wilt is included in the quarantine list of several countries, including Brazil (A1 list since 2018), Egypt (A1 list since 2018), Mexico (quarantine pest since 2018), Israel (quarantine pest since 2009), Japan (quarantine pest since 2022), and China (quarantine pest since 2011). Furthermore, the Asia and Pacific Plant Protection Commission (APPPC) has included the pathogen in the A2 list since 1993. The pathogen has been proposed as a candidate A1 quarantine organism in the EPPO region (EPPO, 2022; Paul & Smith, 1989). Although this disease has been limited to Canada and the United States, it still remains a potential threat anywhere maize is cultivated. Thus, we need to remain alert, monitoring new outbreaks and continuing research on breeding, field testing, and producing new varieties with better disease resistance.

Research over the last 50 years has provided a foundation for knowledge about Goss's wilt that includes a broader understanding of the pathogen and the biotic and abiotic factors that influence disease development. This groundwork and recent technological advancements ensure that in the coming years we will integrate our knowledge into a comprehensive understanding of the ecology of the Goss's wilt pathogen and disease development. Additionally, these technologies will have impacts well beyond Goss's wilt control, for example maize breeding for increased productivity. Whole‐genome sequencing will allow us to identify sequences that can be used in high‐throughput assays to detect and quantify pathogen populations. In addition, whole‐genome sequencing will allow us to predict and test predictions of the genes involved in *C. nebraskensis* pathogenicity. This in turn will allow us to target these mechanisms for disease control. We now have the technology to explore the microbial dark matter consisting of organisms that are not easily culturable. For much of the time since the initial description of Goss's wilt, we were only able to study those microorganisms that could be grown in culture. Metagenomics, transcriptomics, and metabolomics are now being used to help us understand which organisms are present, what they are doing, and how they are doing it. Coupling these tools with spatial and temporal analyses we should be able to predict specific interactions between the Goss's wilt pathogen and maize plants, leading to a more sustainable maize production industry in 21st century agriculture.

## CONFLICT OF INTEREST

The authors declare that the research was conducted in the absence of any commercial or financial relationships that could be construed as a potential conflict of interest.

## Supporting information


**Figure S1** Geographic distribution of *Clavibacter nebraskensis* causing Goss’s wilt of maize in North America. Data obtained from EPPO and CABI databases up to July 2022. Red circles indicate the presence of the pathogen. The source map is from EPPO Global Database https://gd.eppo.int/

## Data Availability

Data sharing is not applicable to this article as no new data were created or analysed.
